# Evaluation of an online training tool for scoring programmed cell death ligand-1 (PD-L1) diagnostic tests for lung cancer

**DOI:** 10.1186/s13000-020-00953-9

**Published:** 2020-04-17

**Authors:** Bharat Jasani, Gudrun Bänfer, Rebecca Fish, Wim Waelput, Yves Sucaet, Craig Barker, Jessica L. Whiteley, Jill Walker, Rudy Hovelinck, Rolf Diezko

**Affiliations:** 1 Pathology Unit, Targos Molecular Pathology GmbH, Kassel, Germany; 2 Advance – Training and Consulting Unit, Targos Molecular Pathology GmbH, Kassel, Germany; 3 Diagnostic Development Unit, Precision Medicine, R&D Oncology, AstraZeneca, Cambridge, UK; 4 Department of Pathology, Vrije Universiteit Brussel (VUB), Universitair Ziekenhuis Brussel (UZ Brussel), Brussels, Belgium; 5 Pathomation, Antwerp, Belgium; 6 Oncology Companion Diagnostics Unit, Precision Medicine, R&D Oncology Unit, AstraZeneca, Cambridge, UK; 7 AstraZeneca BELUX NV/SA, Groot-Bijgaarden, Belgium

**Keywords:** PD-L1, Immunohistochemistry, Training, Scoring, Cut-offs, NSCLC

## Abstract

**Background:**

Numerous studies indicate that higher tumour programmed cell death ligand-1 (PD-L1) expression is associated with greater response to anti-programmed cell death-1 (PD-1)/PD-L1 immunotherapy in non-small cell lung cancer (NSCLC). In the era of precision medicine, there is a need to provide reliable, standardised training for pathologists to improve their accuracy of interpretation and scoring, as the results are used directly to inform clinical decisions. Here we present findings regarding reader reproducibility of PD-L1 tumour cell (TC) staining scoring for NSCLC using a PD-L1 e-trainer tool as part of a PD-L1 immunohistochemistry reader training course.

**Methods:**

The PD-L1 training course was developed based on the use of VENTANA PD-L1 (SP263) and Dako PD-L1 IHC PharmDx 22C3 stained NSCLC samples in combination with a PD-L1 e-trainer tool. Five-hundred formalin-fixed, paraffin-embedded archival samples were obtained from commercial sources and stained for PD-L1. Slides were scored by two expert pathologists, then scanned to produce digital images and re-scored. Thirty-three cases were selected and sorted into three sets: a training set and two self-assessment tests (pre-test and ‘competence’ test). Participants (all selected board-certified pathologists) received face-to-face training including use of an e-trainer tool. Statistical analyses were performed using the competence test set. Overall percentage agreement (OPA) was assessed between the participant pathologists’ registered scores and the reference scores assigned by expert pathologists at clinically relevant PD-L1 cut-offs (≥1%, ≥25% and ≥ 50%).

**Results:**

Seven sessions were held and 69 participant pathologists completed the training. Inter-reader concordance indicated high OPA (85–95%) for PD-L1 TC scoring at clinically relevant cut-offs, with Fleiss’ Kappa > 0.5.

**Conclusions:**

Use of this web-based training tool incorporated into classroom-style training was associated with an overall moderately good level of inter-reader reproducibility at key cut-offs for TC PD-L1 expression testing in NSCLC. Overall, the online training tool offers a means of standardised training for practising pathologists in a clinical setting.

## Background

Anti–programmed cell death-1 (PD-1)/programmed cell death ligand-1 (PD-L1) immunotherapy is well established for the treatment of non-small cell lung cancer (NSCLC). The analysis of tumour PD-L1 expression using immunohistochemistry (IHC) stained samples is a recognised strategy for identifying patients who are most likely to respond to this type of treatment [1].

Multiple IHC tests have been developed and a number of these are commercially available, including the VENTANA PD-L1 (SP263) assay; the Dako PD-L1 IHC PharmDx 22C3 assay; the Dako PD-L1 IHC PharmDx 28-8 assay; and the VENTANA PD-L1 (SP142) assay [1]. In NSCLC, concordance has been demonstrated for tumour cell (TC) staining between the VENTANA PD-L1 (SP263) assay, the Dako PD-L1 IHC PharmDx 28-8 assay and the PD-L1 IHC PharmDx 22C3 assay, indicating that it may be possible to use these assays interchangeably analytically [1].

Depending on the therapeutic regimen and treatment setting, cut-offs of ≥1%, ≥25% and ≥ 50% TC staining have been shown to be clinically relevant in NSCLC [2–4]. It is therefore important that the pathologist is as accurate and consistent as possible when scoring PD-L1 expression and that any avoidable variability (over time, between readers and/or between laboratories) is minimised. Readers, for example, need to be familiar with, and be able to navigate through, tissue and staining artefacts that can lead to errors in scoring and potentially less consistent interpretation.

Lessons learned with other clinical IHC assays are worth considering for PD-L1 testing; for example, in the case of human epidermal growth factor (HER2) testing in breast and gastric cancer, it was found that issues related to interpretation were at least as important for assay concordance as the choice of antibody or the imaging system [5].

In this study, selected board-certified pathologists were invited to participate in a face-to-face training course that incorporated the use of a novel e-trainer tool. We present findings related to the consistency of scoring observed amongst these pathologists in scoring TCs stained for PD-L1 in NSCLC samples following this training.

## Methods

### Aim

To explore reader reproducibility in scoring PD-L1 IHC stained TCs in NSCLC samples at various clinically relevant cut-offs when using the PD-L1 e-trainer tool as part of a PD-L1 IHC reader training course.

### Training course and web-based e-trainer tool development

Five hundred formalin-fixed, paraffin-embedded archival NSCLC samples were obtained from commercial sources (Avaden Biosciences, Seattle, WA, USA; Asterand Bioscience, Royston, UK; BioreclamationIVT, West Sussex, UK). After haematoxylin and eosin staining was used to confirm the histology and presence of > 100 TCs in each sample, approved samples were stained for PD-L1 using both the VENTANA PD-L1 (SP263; Ventana Medical Systems, Inc.) and the Dako PD-L1 IHC PharmDx 22C3 (Agilent Technologies) assays. All staining procedures were carried out according to the manufacturers’ recommended protocols and included a negative reagent control slide. Two qualified pathologists scored the stained tissue samples and assigned a PD-L1 TC percentage score.

Slides were scanned on an Aperio ScanScope® AT system at a 20x optical magnification, resulting in a 0.46 μ/pixel digital image resolution. To avoid the potential introduction of a digital scoring artefact, cases deemed to be of insufficient scanning quality (defined as digital images having a PD-L1 TC percentage score with > 10% deviation of the glass slide score) were excluded. Sixty cases (each stained using both assays and confirmed to have > 100 viable TCs) were selected as a potential teaching set on the basis of providing exposure to a range of difficulties. The selected digital images were uploaded into an online library. To ensure that the conversion to digital format had not introduced any bias, scanned images were re-scored by two expert pathologists to provide a consensus reference score for the digital images for each respective case; the consensus scores on the selected digital images were in each case the same as the scores on the glass slides. Building of the online tool was supported by AstraZeneca, in collaboration with the software development company, Pathomation (Pathomation BVBA, Antwerp, Belgium).

Thirty-three cases were chosen from the online library, all of which were resection samples (20x adenocarcinoma, 9x squamous cell carcinoma, 1x solid carcinoma non-squamous, 1x large cell carcinoma non-squamous, 1x pleomorphic carcinoma non-squamous, 1x non-small cell carcinoma non-squamous). The cases were sorted into three different training sets: 1) demonstration of scoring system set, consisting of 5–10 cases (of which at least 5 were used for demonstration depending on the time available); 2) first self-assessment or pre-test set, consisting of 5 cases; and 3) second self-assessment or ‘competence’ test set, consisting of 18 cases. In the competence test set, the consensus scores for PD-L1 TC expression for samples stained using the VENTANA PD-L1 (SP263) assay were: 3 cases < 1%; 3 cases ≥1–< 25%; 3 cases ≥25–< 50%; 9 cases ≥50%. For samples stained using the Dako PD-L1 IHC PharmDx 22C3 assay, expression was classified as follows: 2 cases < 1%; 6 cases ≥1–< 25%; 4 cases ≥25–< 50%; 6 cases ≥50%.

Laptops, screens, screen set-up and resolution were standardised in order to match as closely as possible across the training sessions. The laptops or PCs all fulfilled at least the minimum requirement for evaluating digital images using the PD-L1 e-trainer tool (i.e. HD-resolution [1920 × 1080 pixels] or higher, with at least 15” screens and a stable internet connection).

### Training course structure and participants

Training meetings were organised and funded by AstraZeneca; the meetings were open to board-certified pathologists in the relevant regions who were actively involved in PD-L1 testing. Training meetings were held in Hong Kong, Germany, the USA, Panama and South Korea. The general structure of each meeting comprised: a lecture session; a review of example PD-L1 IHC images (NSCLC); an initial pre-test self-assessment session, followed by discussion of results; and then self-assessment of a larger competence testing sample set.

For the competence test, the participant pathologists were allowed to select whether to score samples stained using either or both of the Dako PD-L1 IHC PharmDx 22C3 assay and the VENTANA PD-L1 (SP263) assay. The training was designed in this way to provide participating pathologists with an opportunity to score the samples in accordance with their local practice.

### Data analysis

Results from scoring the competence test set were analysed to determine the overall percentage agreement (OPA, %), mean score, and standard deviation (SD). OPA was assessed between individual participant pathologist’s registered scores (reported as raw percentages) and the reference scores assigned by expert pathologists using three different PD-L1 cut-off levels, ≥1%, ≥25% or ≥ 50% of TCs positive for PD-L1 expression in a given NSCLC sample. PD-L1 cut-off levels and scoring criteria were the same for samples stained with either of the assays. Fleiss’ Kappa values of the inter-reader reliability of the scoring by the 69 participant pathologists in classifying each case as PD-L1 high or low/negative were calculated using R version 3.5.1. The following criteria were applied to interpret Fleiss’ Kappa values of inter-reader reliability: poor, < 0; slight, 0.01–0.20; fair, 0.21–0.40; moderate, 0.41–0.60; substantial, 0.61–0.80.

## Results

Seven training sessions were held across five locations: Hong Kong (× 2; *n* = 8 and *n* = 15), Germany (× 2; *n* = 6 and *n* = 8), USA (× 1; *n* = 11), Panama (× 1; *n* = 12) and South Korea (× 1; *n* = 9). A total of 69 pathologists completed the training for scoring PD-L1 expression in NSCLC samples.

In total, VENTANA PD-L1 (SP263) assay stained samples were scored by 59 pathologists and the Dako PD-L1 IHC PharmDx 22C3 assay stained samples were scored by 15 pathologists. From these groups, five pathologists scored both the PD-L1 (SP263) and the Dako PD-L1 IHC PharmDx 22C3 assays (participant pathologists’ choice, based on their local practice).

### Inter-reader agreement

**Fig. 1:**
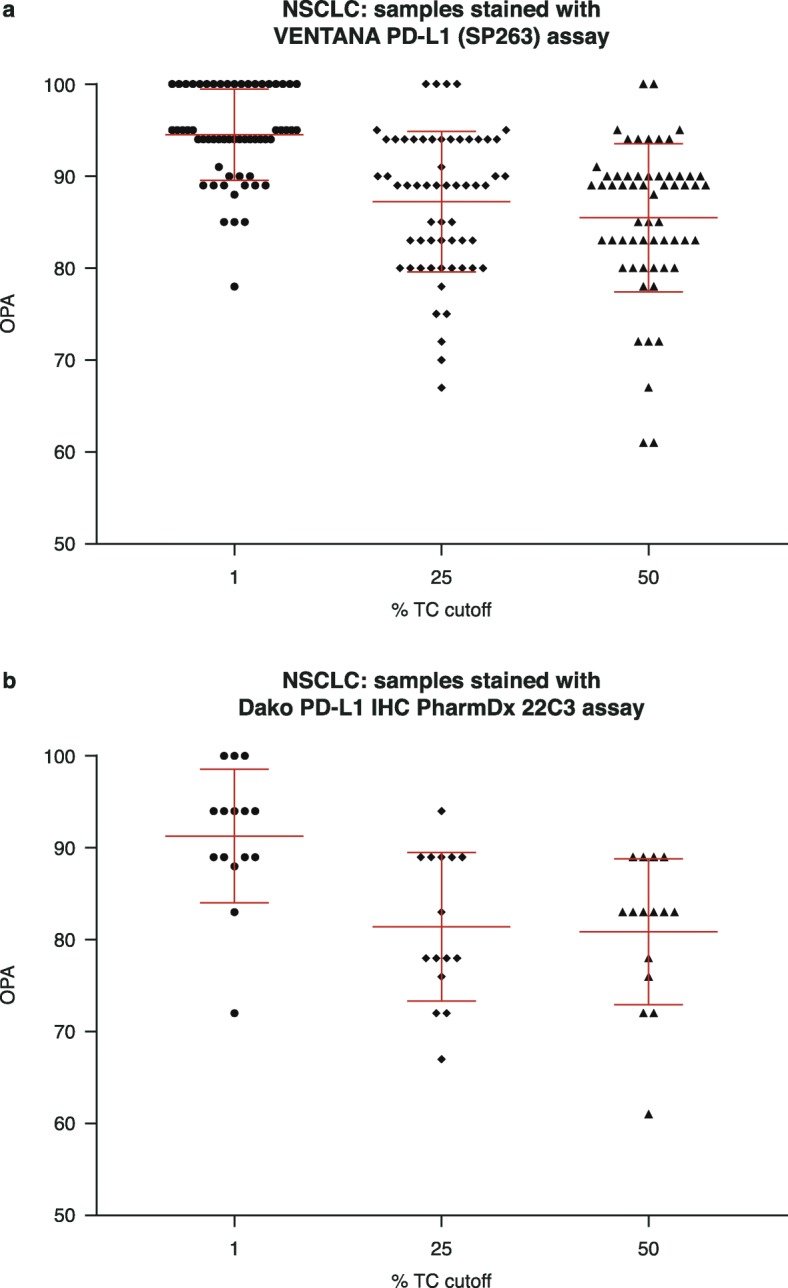
Overall percentage agreement (TCs) between board-certified pathologists’ scoring for NSCLC using (**a**) the VENTANA PD-L1 (SP263) assay (*n* = 59) and (**b**) the Dako PD-L1 IHC PharmDx 22C3 assay (*n* = 15), NSCLC, non-small cell lung cancer; OPA, overall percentage agreement; TC, tumour cell. Red lines represent standard deviation. Assays were carried out according to manufacturers’ recommended protocols

After training using the VENTANA PD-L1 (SP263) assay stained slides, mean OPA ranged from 85% (95% confidence interval [CI]: 83–88%) using the ≥50% TC cut-off, to 95% (95% CI: 93–96%) with the ≥1% TC cut-off (*n* = 59; Fig. 1a). After training using the Dako PD-L1 IHC PharmDx 22C3 assay stained slides, mean OPA was 81% (95% CI: 76–85%) at the ≥50% TC cut-off and 91% (95% CI: 87–95%) at the ≥1% TC cut-off, with best agreement at a ≥ 1% TC cut-off (*n* = 15; Fig. 1b).

**Fig. 2:**
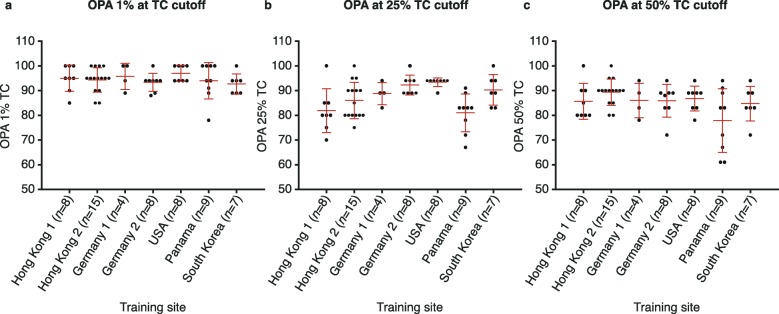
Overall percentage agreement for the (**a**) 1% TC cut-off, (**b**) 25% TC cut-off and (**c**) 50% TC cut-off (TCs) between geographical regions for NSCLC samples stained using the VENTANA PD-L1 (SP263) assay (*n* = 59), NSCLC, non-small cell lung cancer; OPA, overall percentage agreement; TC, tumour cell. Red lines represent standard deviation. Assay was carried out according to manufacturers’ recommended protocols

Fleiss’ Κappa analysis of inter-reader reliability indicated that, at all three TC cut-offs (≥1%, ≥25% and ≥ 50%), the scoring was moderately reliable (Κ 0.581, 0.556 and 0.535, respectively) for classifying cases as PD-L1 high or low/negative. Performance at different training sites was of a generally similar standard (Fig. 2).

## Discussion

The findings from our ‘classroom-style’ training provide the ‘proof of principle’ evidence that the combination of ‘hands-on’ training from an experienced trainer and a web-based e-trainer tool is capable of delivering an acceptable level of inter-reader reproducibility for PD-L1 IHC TC scoring in NSCLC at ≥1%, ≥25% and ≥ 50% cut-offs using the VENTANA PD-L1 (SP263) assay or the Dako PD-L1 IHC PharmDx 22C3 assay.

The OPAs (85–95%) between different participant pathologists were comparable to those observed in other publications achieved via light microscope evaluation (SP263 96.7% OPA at the ≥25% cut-off and 22C3 92.7% OPA at the ≥50% cut-off) [6, 7]; these findings imply similar results when scoring stained tissue from either glass slides or via the digital images. Fleiss’ Κappa analysis of inter-reader reliability indicated the scoring to be moderately reliable for classifying cases as PD-L1 high or low/negative at all three cut-offs. Given that the samples in this study included several challenging cases and encompassed a range of cut-offs (selected to provide examples of the full range of PD-L1 expression observed in patients with NSCLC) and sample types, representative of a real-life clinical setting, these findings concur well with the inter-reader concordance rates and tests of reproducibility that have been reported as achievable for PD-L1 determination [8–10]. In a study by Scheel et al., scoring of PD-L1-positive TCs using integrated dichotomous proportion cut-offs (≥1%, ≥5%, ≥10% and ≥ 50%) showed a good concordance coefficient (Light’s Κappa 0.6–0.8) [9]. In our experience, the higher the threshold used for categoric scoring, the greater the likelihood of the level of variability observed with respect to the raw scores derived for these cases. We hypothesise that this variability is probably reflective of the greater degree of effort necessary for accurate counting of the total number of viable TCs and similarly, the total number of PD-L1 stained TCs, especially in larger resection specimens. There is a tendency, therefore, for pathologists to rely more on ‘guestimates’ based on pattern recognition than the actual counting of the TCs involved. The lower inter-rater variability in the published studies may relate to greater stringency used in accounting for the relevant cells.

The following factors may have contributed to the observed levels of inter-reader agreement in these training sessions. Guidance from an experienced trainer, linked to the use of high-quality, standardised digital image viewing equipment was used. The e-trainer tool also included a screen quality assessment whereby the participant pathologists confirmed their ability to distinguish between anthracotic pigment and 3,3-Diaminobenzidine (DAB) staining in a series of reference images as shown on their monitor. Finally, all the participants were guided by the online tool to provide feedback on the serviceability of the tool, highlighting good levels of image manipulation and responsiveness.

Post-training continued access to online training may be of further benefit. The online PD-L1 e-trainer tool is freely available. Up-to-date information and an open access trial version of the tool can be found on the IDPDL1 website: https://www.idpdl1.com/quality-and-training.html.

The facility to revisit training materials subsequent to the initial training is important, as continual retraining or recalibration is key to maintaining good levels of inter-reader and inter-laboratory consistency and in minimising any intra-reader ‘drift’. In addition, readers who score slides infrequently are less likely to be internally consistent and have a greater chance of mis-scoring the samples.

The significance of the results presented here is associated with certain limitations. Firstly, a relatively small sample of invited board-certified pathologists were included for training and they were not assessed at baseline for their ability to interpret and score PD-L1 staining; therefore, it is not possible to assess the true impact of the training at the wider community level. We have a plan to address this limitation as part of a future training programme. Secondly, training was carried out on scanned images displayed on a screen rather than on glass slides, and no comparison was made of the training experience or effectiveness of training between the two modes of analyses. However, the expert pathologists did assess the scoring efficacy between the glass and digital image-based approaches and they were found to be comparable. Nevertheless, the digital image-based method of scoring differs from routine clinical practice, which is largely based on the use of glass slides. PD-L1 stained slides were only scanned at 20x optical magnification. In some critical cases this may be insufficient (e.g. when assessing weak membranous staining requiring a more detailed assessment at a higher optical magnification of 40x). Fourthly, as with any digital image, a key difference from glass slides is the inability to move around the sample quickly and smoothly to assess areas representative of the overall staining pattern. This is particularly crucial for cases showing heterogeneous patterns of staining. However, it is believed that users are likely to become more capable of overcoming this limitation through regular working with digital images over a period of time. Finally, feedback from the participant pathologists indicated that a possible area for future improvement of the PD-L1 e-trainer tool would be the inclusion of a greater proportion of NSCLC biopsy cases. This would provide a training experience on a sample type most frequently assessed in routine diagnostic practice.

Nevertheless, overall, the online training tool offers a means of standardised training for pathologists using specimen material encountered in a routine clinical setting. The acceptable levels of inter-reader reproducibility at key cut-offs for PD-L1 expression testing in our study, using the VENTANA PD-L1 (SP263) and the Dako PD-L1 IHC PharmDx 22C3 assays, provide the ‘proof of principle’ evidence that the PD-L1 e-trainer tool can be used more frequently in the future to improve and confirm inter-reader consistency for TC PD-L1 scoring in NSCLC.

## Data Availability

The detailed data underlying the findings described in this manuscript may be obtained in accordance with AstraZeneca’s data sharing policy described at: https://astrazenecagrouptrials.pharmacm.com/ST/Submission/Disclosure.

## Abbreviations

CI: Confidence interval
DAB: 3,3-Diaminobenzidine
HER2: Human epidermal growth factor 2
IHC: Immunohistochemistry
NSCLC: Non-small cell lung cancer
OPA: Overall percentage agreement
PD-1: Programmed cell death-1
PD-L1: Programmed cell death ligand-1
SD: Standard deviation
TC: Tumour cell
